# Family-Based Dietary Counselling in Pediatric Obesity: A Proposed System-Oriented Framework Integrating Home, School, and Social Environments

**DOI:** 10.3390/nu18121949

**Published:** 2026-06-17

**Authors:** Paulina Metelska, Agnieszka Kozioł-Kozakowska

**Affiliations:** 1 Department of Public Health & Social Medicine, Faculty of Health Sciences with the Institute of Maritime and Tropical Medicine, Medical University of Gdansk, 80-211 Gdansk, Poland; 2 Department of Pediatrics, Gastroenterology and Nutrition, Jagiellonian University Medical College, 30-663 Cracow, Poland; agnieszka.koziol-kozakowska@uj.edu.pl

**Keywords:** pediatric obesity, childhood obesity, dietary counselling, nutrition education, family-based intervention, behavioral treatment, food environment, lifestyle intervention, eating behavior, public health

## Abstract

**Background/Objectives**: Childhood obesity is a complex, multifactorial condition that requires comprehensive and sustained interventions. Despite the central role of dietary modification in obesity management, current approaches to dietary counselling remain heterogeneous and often fail to account for the broader environmental and social determinants of eating behavior. In pediatric populations, dietary habits are strongly influenced by family dynamics, home food environments, school settings, and peer interactions, highlighting the need for system-oriented intervention models. **Methods**: This structured narrative review with conceptual framework development presents an integrative framework for dietary counselling in pediatric obesity, combining evidence-based nutritional strategies with behavioral and environmental approaches. The paper synthesizes current literature on early-life habit formation, family-based behavioral treatment, feeding practices, and environmental determinants of dietary behavior. **Results**: The proposed framework emphasizes the role of the family as the primary therapeutic unit and highlights the importance of modifying the home food environment and implementing gradual, achievable changes through the “small steps” approach. A structured, visit-based model of dietary counselling is introduced, integrating dietary assessment, patient education, and behavioral strategies. Additionally, the influence of external environments—including schools, peer groups, and public health systems—is considered to provide a comprehensive understanding of factors shaping dietary behaviors in children. **Conclusions**: The proposed system-oriented framework offers practical guidance for clinicians and public health practitioners and supports the development of more effective and sustainable interventions. Integrating individual, family, and environmental perspectives may improve adherence to dietary recommendations and enhance long-term outcomes in pediatric obesity management.

## 1. Introduction

Childhood obesity represents one of the most pressing public health challenges of the 21st century, with prevalence rates increasing rapidly across both developed and developing countries. According to the World Health Organization, the number of children and adolescents living with overweight and obesity has risen dramatically over recent decades, contributing to a growing burden of noncommunicable diseases, including type 2 diabetes, cardiovascular disorders, and metabolic syndrome [1].

Obesity is a multifactorial and chronic disease that arises from a complex interplay of biological, behavioral, environmental, and social determinants. While advances in pharmacotherapy, including glucagon-like peptide-1 receptor agonists (GLP-1 RAs), have expanded treatment options, lifestyle interventions—particularly dietary modification—remain a cornerstone of obesity management, especially in pediatric populations. However, despite the central role of nutrition in obesity care, there is currently no universally standardized approach to dietary counselling, and its implementation in clinical practice remains inconsistent [2].

In children, dietary behaviors are not formed in isolation but are embedded within a broader ecological system that includes the family environment, school setting, peer influences, and wider socio-cultural context. Evidence consistently highlights the critical role of parents and caregivers as primary agents shaping children’s eating behaviors through mechanisms such as behavioral modeling, food availability, and feeding practices. In parallel, external environments—including schools and peer groups—further reinforce or undermine these behaviors, contributing to the complexity of effective intervention [3,4].

Current clinical approaches often focus on individual-level dietary recommendations without sufficiently addressing the systemic and environmental factors that sustain obesogenic behaviors. Family-Based Behavioral Treatment (FBT) has emerged as a gold standard in pediatric obesity management, emphasizing the need for simultaneous lifestyle modification within the entire household. Nevertheless, translating such approaches into routine clinical practice remains challenging due to time constraints, variability in professional training, and limited integration across healthcare and community systems [5].

Although FBT provides a strong evidence-based foundation for pediatric obesity treatment, its implementation in routine practice may be constrained by limited time, workforce capacity, family heterogeneity, and variable access to multidisciplinary care. Moreover, many counselling models remain focused primarily on the family and home environment, while school food environments, peer influences, neighborhood conditions, public institutions, and broader food systems are often addressed only indirectly. This creates a practical gap between evidence-based behavioral treatment and the complex social environments in which children’s eating behaviors occur [5,6,7].

Given these challenges, there is a need for integrative frameworks that combine evidence-based dietary counselling with a system-oriented perspective, accounting for the multiple environments influencing a child’s behavior. Such frameworks should not only provide nutritional guidance but also incorporate behavioral strategies, family dynamics, and environmental modifications to enhance the sustainability of lifestyle changes.

The proposed framework is grounded in three complementary perspectives: ecological systems theory, which emphasizes the nested influence of family, school, community, and policy environments on child development; social learning theory, which explains how children acquire eating behaviors through observation and modeling; and systems-based models of obesity, which conceptualize obesity as the result of interacting biological, behavioral, environmental, and policy-level determinants [2,7,8,9].

The aim of this paper is to present a system-oriented framework for dietary counselling in pediatric obesity, integrating family-based approaches with environmental and institutional factors, and to provide practical guidance for implementing these strategies in clinical and public health settings.

## 2. Materials and Methods

This paper adopts a narrative review approach combined with a conceptual framework development to synthesize current knowledge on dietary counselling in pediatric obesity and to propose a system-oriented model for clinical practice.

The review was conducted as a structured narrative review with conceptual framework development. This approach was selected because the aim of the paper was not to estimate intervention effects quantitatively, but to synthesize evidence from clinical guidelines, behavioral science, dietary counselling literature, and public health frameworks into a practical model for use in pediatric obesity care [10].

A targeted literature search was conducted using databases including PubMed, Scopus, and Google Scholar, as well as reports and guidelines from international organizations such as the World Health Organization. The search focused on studies published in English within the last 10–15 years, with particular emphasis on pediatric populations.

Key search terms included combinations of: pediatric obesity, childhood obesity, dietary counselling, nutrition education, family-based interventions, behavioral treatment, food environment, lifestyle interventions, cultural perceptions of obesity; weight stigma; single-parent families; low-resource settings; socioeconomic disparities; school food environment; neighborhood food environment; food insecurity; built environment; health equity; ecological model; systems approach; behavior change framework. Additional relevant publications were identified through manual screening of reference lists.

The search strategy was organized around four thematic domains: (1) pediatric obesity treatment and dietary counselling; (2) family-based behavioral treatment and parental feeding practices; (3) environmental and social determinants of children’s eating behaviors, including home, school, peer, and community environments; and (4) theoretical and conceptual models relevant to systems-oriented health behavior change. In addition to international guidelines, selected national and regional guidelines were reviewed when they addressed multidisciplinary pediatric obesity care, family-based interventions, or dietary counselling implementation.

Titles and abstracts were screened for relevance to the review objectives. Full texts were then reviewed and information was extracted narratively into thematic categories, including study population, intervention setting, family involvement, behavioral strategies, environmental components, and implications for dietary counselling. The synthesis was performed by the authors through iterative discussion, with the aim of identifying recurring components that could inform the proposed framework.

Original research studies (including randomized controlled trials and observational studies), systematic reviews, and clinical guidelines were all considered. Particular attention was given to evidence related to family-based behavioral treatment, environmental determinants of eating behavior, and practical tools used in dietary assessment and counselling.

The selection of literature was guided by relevance to the topic rather than strict inclusion and exclusion criteria, consistent with a narrative review methodology. The identified evidence was then synthesized and integrated with clinical practice insights to develop a structured, system-oriented framework for dietary counselling in children with obesity.

## 3. Results

### 3.1. The Role of Early-Life Nutrition and Habit Formation

The development of dietary behaviors begins early in life and plays a crucial role in shaping long-term health outcomes. Evidence indicates that eating patterns established during infancy and early childhood tend to persist into adolescence and adulthood, influencing the risk of obesity and other diet-related diseases [11].

The period of complementary feeding represents a critical window for the formation of taste preferences and dietary habits. Early exposure to a variety of foods, particularly vegetables and minimally processed products, has been associated with greater acceptance and healthier dietary patterns later in life. Conversely, frequent exposure to energy-dense, highly palatable foods may predispose children to preferences that promote excessive energy intake [11].

Parents and caregivers play a central role in this process, acting as primary models of eating behavior. Through observational learning, children adopt not only food choices but also attitudes toward eating, including emotional responses and regulatory patterns. For example, the use of food as a reward or a coping mechanism for stress has been linked to the development of emotional eating behaviors and impaired self-regulation of hunger and satiety [12].

Importantly, the establishment of healthy dietary habits extends beyond knowledge acquisition and requires consistent environmental reinforcement. Structured meal patterns, repeated exposure to healthy foods, and supportive feeding practices are key determinants of sustainable behavior change. These findings highlight the need for early, family-centered interventions that promote positive dietary behaviors from the earliest stages of life.

### 3.2. Family as the Core Therapeutic Environment

Childhood obesity cannot be effectively addressed without considering the family context in which dietary behaviors are formed and maintained. The family represents the primary environment influencing a child’s lifestyle, shaping not only food choices but also behavioral norms, emotional regulation, and attitudes toward health.

#### 3.2.1. Feeding Styles and Their Impact

Feeding styles are defined as patterns of parental attitudes and behaviors related to child feeding and are typically categorized as authoritative, authoritarian, permissive, or neglectful. Recent evidence confirms that parental feeding styles significantly influence children’s dietary behaviors and obesity risk, with authoritative approaches consistently associated with more favorable outcomes. Similarly, permissive feeding styles, marked by a lack of boundaries and structure, have been associated with increased consumption of energy-dense foods and irregular eating patterns [13,14].

In contrast, an authoritative feeding style—balancing structure with responsiveness—is considered the most beneficial for supporting healthy eating behaviors. This approach promotes autonomy while maintaining appropriate guidance, facilitating the development of internal self-regulation mechanisms [15].

#### 3.2.2. Behavioral Modeling and Emotional Context of Eating

Children learn eating behaviors primarily through observation. Parental modeling influences not only the types of foods consumed but also the context in which eating occurs. For instance, regular family meals have been associated with improved dietary quality, while chaotic eating patterns may contribute to irregular intake and overeating [15,16].

The emotional context of eating is equally important. When food is used as a reward, punishment, or coping strategy, children may develop maladaptive relationships with food, increasing the risk of emotional eating and long-term weight-related problems [17].

#### 3.2.3. Genetic and Environmental Interactions

While genetic predisposition contributes to obesity risk, environmental and behavioral factors play a decisive role in its expression. Children growing up in households with obesogenic environments—characterized by high availability of processed foods, low physical activity, and inconsistent routines—are significantly more likely to develop obesity, regardless of genetic background [18].

These findings underscore the importance of addressing the family system as a whole in therapeutic interventions, rather than focusing solely on the individual child.

### 3.3. The Home Food Environment

The home food environment constitutes a critical determinant of children’s dietary behaviors and plays a central role in both the development and management of obesity.

#### 3.3.1. Food Availability and Accessibility

The types of foods available within the household strongly influence children’s consumption patterns. High availability of energy-dense, nutrient-poor foods—such as sweets, sugary beverages, and processed snacks—has been consistently associated with increased intake of these products. Conversely, easy access to fruits, vegetables, and minimally processed foods supports healthier dietary choices [19].

Importantly, availability is closely linked to accessibility. Foods that are visible, easily reachable, and ready to consume are more likely to be selected, particularly by children with limited self-regulation capacities [19].

#### 3.3.2. Environmental Cues and Eating Behavior

The home environment is rich in cues that can trigger eating behavior independently of physiological hunger. These cues include visual exposure to food, habitual eating contexts (e.g., eating while watching television), and social norms within the household.

The concept of an “obesogenic environment” highlights how constant exposure to high-calorie foods creates a mismatch between environmental stimuli and biological regulation mechanisms. Children exposed to such environments may experience difficulty adhering to dietary recommendations, even when motivated to change [7].

#### 3.3.3. Household-Level Interventions

Modifying the home environment is a key strategy in dietary counselling. Effective interventions include: reducing the availability of highly processed foods, restructuring food storage to promote healthier choices, planning meals and shopping lists to align with dietary goals and creating consistent meal routines. Rather than relying solely on individual self-control, these strategies aim to reshape the environment in a way that naturally supports healthier behaviors [20].

### 3.4. Family-Based Behavioral Treatment (FBT)

Family-Based Behavioral Treatment (FBT) is widely recognized as one of the most effective approaches for managing pediatric obesity. This model emphasizes the involvement of the entire family in the process of lifestyle modification, rather than focusing exclusively on the child. FBT integrates principles from behavioral psychology, including goal setting, self-monitoring, reinforcement, and problem-solving. Parents are positioned as agents of change, responsible for modifying the home environment, modeling healthy behaviors, and supporting the child’s efforts [21].

Evidence from randomized controlled trials indicates that FBT leads to significant improvements in weight-related outcomes, dietary behaviors, and physical activity levels. Importantly, these effects are often sustained over time, highlighting the long-term benefits of family-centered interventions [6].

A key advantage of FBT is its systemic perspective, which acknowledges that sustainable change requires alignment across multiple domains of daily life. By addressing family routines, communication patterns, and environmental factors, this approach increases the likelihood of lasting behavioral change [5].

### 3.5. The “Small Steps” Approach in Dietary Counselling

The “small steps” approach represents a practical and effective strategy for implementing dietary and lifestyle changes in pediatric obesity.

#### 3.5.1. Theoretical Background

This approach is grounded in behavioral theories, including cognitive–behavioral therapy (CBT) and the transtheoretical model of behavior change. These frameworks emphasize the importance of gradual, stage-based change and the need to tailor interventions to the individual’s readiness and capacity [9,22].

#### 3.5.2. Practical Implementation

In clinical practice, the small steps approach involves introducing one or two achievable changes at a time, rather than attempting comprehensive lifestyle modification. Goals are typically formulated according to the SMART criteria (Specific, Measurable, Achievable, Relevant, Time-bound), ensuring clarity and feasibility.

Examples of initial changes may include reducing the consumption of sugary beverages, establishing regular meal times, or increasing daily physical activity through simple, enjoyable activities.

#### 3.5.3. Clinical Advantages

Gradual change reduces the risk of overwhelm and increases adherence to recommendations. Early successes enhance motivation and self-efficacy, creating a positive feedback loop that supports further progress.

Additionally, this approach acknowledges the non-linear nature of behavior change, normalizing setbacks and framing them as opportunities for learning rather than failure [9,22].

### 3.6. Assessment Tools in Dietary Counselling

A comprehensive assessment of dietary intake is a fundamental component of effective nutritional counselling. Accurate identification of eating patterns allows clinicians to tailor interventions to the individual needs, preferences, and environmental context of the child and their family.

In addition to anthropometric outcomes, counselling should monitor behavioral and contextual indicators, including meal frequency, breakfast consumption, intake of sugar-sweetened beverages, fruit and vegetable intake, frequency of ultra-processed snack consumption, screen time, sleep duration, physical activity, school food purchases, and caregiver-reported barriers to change. These indicators may be more sensitive to early behavioral progress than body weight alone [5,23,24].

#### 3.6.1. Comprehensive Dietary Assessment Methods

Several validated methods are available for assessing dietary intake. The 24 h dietary recall provides detailed information on recent consumption and is commonly used in both clinical and research settings. Food Frequency Questionnaires (FFQs) allow for the evaluation of habitual intake over a longer period, while food diaries offer insights into daily eating patterns, portion sizes, and contextual factors such as timing and emotional triggers. Although these methods provide valuable data, they can be time-consuming and may be subject to reporting bias, particularly in pediatric populations where parental reporting is often required [25].

#### 3.6.2. Screening Tools in Clinical Practice

In routine clinical settings, shorter screening tools are often more feasible. Brief instruments, such as structured dietary questionnaires or rapid assessment tools, can identify key risk behaviors, including low fruit and vegetable intake, high consumption of sugary beverages, and frequent intake of processed foods [23].

Short consultations—lasting as little as 3–5 min—can still be effective when structured tools are used to guide the conversation. These tools are particularly useful in primary care settings, where time constraints limit the use of comprehensive assessments [23].

#### 3.6.3. Application in Pediatric Populations

When working with children, dietary assessment should extend beyond individual intake to include family practices and environmental influences. Evaluating meal patterns, food availability at home, and parental behaviors provides a more complete understanding of the factors driving dietary habits [24].

Importantly, assessment should be conducted in a non-judgmental and supportive manner. The primary goal is not to evaluate compliance but to identify opportunities for realistic and achievable changes, consistent with a patient-centered approach [26].

### 3.7. Translating Assessment into Dietary Education

Following the initial assessment, the focus shifts to translating identified dietary patterns into practical, individualized recommendations. Effective dietary education should be tailored to the cognitive and developmental level of the child, as well as to the resources and constraints of the family [5,23].

Visual tools and simplified frameworks—such as food-based dietary guidelines or plate models—can facilitate understanding and support behavior change. However, the effectiveness of these tools depends largely on their contextualization within the family’s daily routine [27,28].

Rather than providing generic advice, clinicians should prioritize the identification of one or two key areas for modification. This targeted approach aligns with behavioral change principles and increases the likelihood of successful implementation.

Importantly, education should not be limited to knowledge transfer but should also include skill-building, such as meal planning, label reading, and food preparation. Engaging both the child and caregivers in the educational process enhances motivation and reinforces behavioral change within the home environment [5,23].

### 3.8. A Structured Model of Dietary Counselling: A System-Oriented Framework

**Figure 1:**
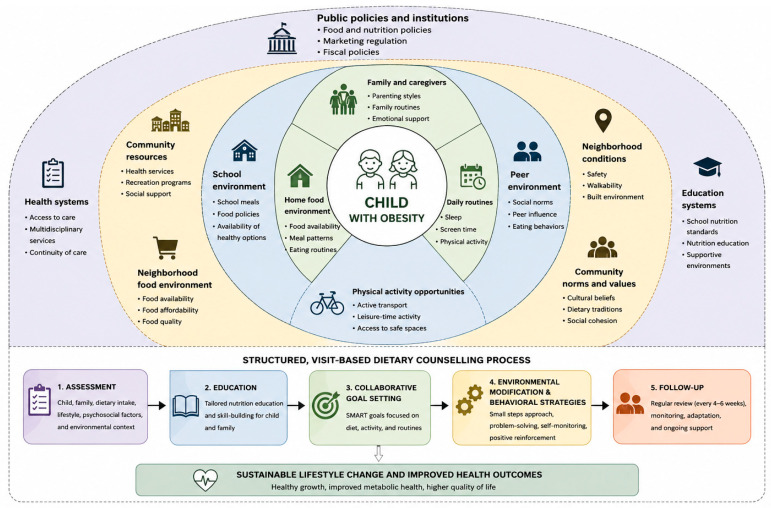
System-oriented framework for dietary counselling in pediatric obesity. The framework places the child within nested and interacting environments: family and home food environment, school and peer context, community and neighborhood conditions, and public institutions and food policy. Dietary counselling is conceptualized as a structured, visit-based process integrating assessment, education, collaborative goal setting, behavioral strategies, and follow-up.

Based on the synthesis of current evidence and clinical practice insights, a structured, visit-based model of dietary counselling can be proposed for the management of pediatric obesity (Figure 1).

#### 3.8.1. Visit-Based Intervention Model

The counselling process may be organized into a series of structured visits, each focusing on specific aspects of dietary behavior and lifestyle modification.

The proposed structured visit-based counselling model is presented in Table 1.

The table presents a practical framework for organizing dietary counselling visits in pediatric obesity care, including suggested assessment tools, counseling targets, behavioral strategies, and follow-up components. The proposed model integrates nutritional, behavioral, and environmental approaches and may be adapted to different clinical and community settings. The proposed model was developed based on current pediatric obesity guidelines, family-based behavioral treatment principles, and behavior change frameworks [5,6,23,29].

The proposed model is intended as a flexible clinical framework rather than a rigid protocol. Depending on healthcare setting, family structure, available resources, cultural context, and obesity severity, individual components may be expanded, combined, or adapted. Importantly, the framework may also be applied in single-parent households and other non-traditional family structures by focusing on the primary caregivers and environmental determinants shaping the child’s eating behaviors.

Physical activity counselling within the proposed framework should emphasize reducing sedentary time, increasing enjoyable daily movement, and identifying feasible family-based activities rather than prescribing exercise in a punitive or weight-centered manner. For younger children, play-based movement and active transport may be prioritized, whereas adolescents may benefit from autonomy-supportive planning that accounts for preferences, peer context, and perceived barriers. The primary goal is to support sustainable lifestyle integration rather than short-term exercise compliance [5,30,31].

#### 3.8.2. Integration with Behavioral Strategies

The effectiveness of dietary counselling is enhanced when combined with behavioral techniques, including goal setting, self-monitoring, and reinforcement. Collaborative goal-setting—where the family actively participates in defining targets—has been shown to improve adherence and outcomes.

Limiting the number of goals introduced at each stage is essential to avoid cognitive overload and to support sustained engagement [22,29].

#### 3.8.3. Adaptability and Personalization

The proposed model is inherently flexible and can be adapted to different clinical settings, age groups, and levels of obesity severity. Personalization is key, as interventions must align with the family’s cultural context, socioeconomic conditions, and readiness for change.

This adaptability makes the model applicable not only in specialized obesity clinics but also in primary care and community-based settings [5,23]. However, the implementation of the proposed framework may require varying levels of human and organizational resources depending on the healthcare setting. Comprehensive multidisciplinary delivery involving physicians, dietitians, psychologists, physical activity specialists, and social support services may improve effectiveness but can also increase operational costs and pose challenges for long-term sustainability. Therefore, local adaptation and prioritization of available resources are likely to be essential for successful implementation [5,32].

### 3.9. The Role of the School Environment

The school environment plays a significant role in shaping children’s dietary behaviors, as it represents a major context for daily food consumption outside the home [33]. School meals, access to vending machines, and opportunities to purchase food independently can all influence dietary intake. In some cases, children may consume multiple main meals—both at school and at home—leading to excessive energy intake [33].

Assessment of school-related eating behaviors should therefore be an integral part of dietary counselling. Interventions may include collaboration with parents to plan school meals, guidance on managing food purchases, and education on making healthier choices within the school setting [34].

Additionally, schools have the potential to serve as platforms for health promotion through structured nutrition education and supportive food policies [33].

### 3.10. Peer Influence and Social Context

During childhood and adolescence, peer relationships exert a strong influence on behavior, including dietary choices. Social norms, group dynamics, and the desire for acceptance can significantly shape eating patterns [30].

Children may adopt dietary behaviors that align with their peer group, even when these behaviors are inconsistent with health recommendations. For example, shared consumption of energy-dense snacks or sugary beverages may reinforce unhealthy habits [30].

At the same time, children with obesity may experience stigma, social exclusion, or bullying, which can negatively impact psychological well-being and contribute to maladaptive eating behaviors such as emotional eating or secretive eating [35].

Addressing the social context of eating is therefore essential in dietary counselling. Interventions should include strategies to strengthen the child’s self-efficacy, promote positive peer interactions, and, where possible, engage group-based approaches to behavior change [5].

### 3.11. Community and Neighborhood Context

Beyond the home and school environments, neighborhood and community conditions can influence dietary behaviors and obesity risk. Access to affordable healthy foods, density of fast-food outlets, walkability, availability of safe recreational spaces, transportation barriers, and neighborhood safety may all shape families’ ability to implement dietary and lifestyle recommendations. In low-resource settings, counselling that assumes easy access to fresh foods, safe outdoor activity, or stable household routines may be unrealistic. Therefore, dietary counselling should include a brief assessment of community-level barriers and should help families identify feasible alternatives within their actual environment [7,36,37].

### 3.12. The Role of Public Institutions

Effective obesity prevention and management require coordinated action at the population level. Public institutions play a key role in shaping food environments and supporting healthy behaviors [7].

At the institutional level, dietary counselling is more likely to be effective when individual recommendations are supported by consistent food policies and accessible services. Examples include school nutrition standards, restrictions on marketing of unhealthy foods to children, fiscal policies targeting sugar-sweetened beverages, and public financing of multidisciplinary obesity care. These measures do not replace clinical counselling, but they can reduce the environmental burden placed on children and families [2,38,39].

Policy interventions—such as regulating food marketing, implementing taxes on sugar-sweetened beverages, and establishing nutritional standards in schools—have been shown to influence dietary patterns at the population level [39].

In addition, public health campaigns and school-based programs can increase awareness and provide practical tools for healthy eating. Ensuring equitable access to healthy foods is particularly important, as socioeconomic disparities significantly affect dietary behaviors and obesity risk [40].

A multisectoral approach, involving healthcare systems, educational institutions, and policy makers, is essential for creating environments that support individual and family-level interventions [5,23].

### 3.13. Case Example: A Local Prevention Program

Local, community-based programs can provide valuable support for families managing childhood obesity. Such programs often offer multidisciplinary care, including dietary counselling, psychological support, and physical activity interventions [41].

An example of this approach is a structured prevention program targeting children at key developmental stages, combining early detection with long-term, family-oriented support. These programs demonstrate that integrating clinical care with community resources can enhance accessibility and improve outcomes [42].

While local initiatives may vary in scope and design, they highlight the importance of bridging gaps between healthcare systems and community environments.

## 4. Discussion

This paper proposes a system-oriented framework for dietary counselling in pediatric obesity, integrating individual, family, and environmental determinants of behavior. Such an approach is consistent with ecological and systems-based models of obesity, which emphasize the interaction between biological, behavioral, and environmental factors in shaping health outcomes [7].

The main contribution of this review is the integration of dietary counselling, behavioral strategies, and environmental assessment into a single practical framework. While existing approaches such as FBT provide strong evidence for involving the family in pediatric obesity treatment, the present framework extends this perspective by explicitly incorporating school eating behaviors, peer influence, neighborhood constraints, and public health systems into the counselling process. This may help clinicians move from general dietary advice toward a structured assessment of the environments that facilitate or hinder behavior change [5,6,7].

The findings emphasize that effective dietary interventions cannot rely solely on individual-level recommendations. Instead, they must address the broader context in which eating behaviors occur, including family dynamics, home food environments, school settings, and social influences.

Systematic reviews indicate that parent-focused interventions can lead to measurable improvements in children’s BMI and dietary behaviors, further supporting the central role of the family in obesity management [15,17]. Importantly, family-based approaches should not be interpreted exclusively within the context of traditional two-parent households. Contemporary family structures are diverse and may include single-parent families, blended families, multigenerational households, shared custody arrangements, or caregivers other than parents. In such contexts, the term “family” should be understood functionally, referring to the person or persons who shape the child’s food environment, routines, and access to care. Therefore, the proposed framework should be applied flexibly and adapted to the social, economic, and cultural realities of each household [2,5].

This reinforces the need to move beyond individual-level counselling toward integrated, system-level interventions.

The integration of behavioral strategies—such as the small steps approach and family-based treatment—enhances the feasibility and sustainability of interventions. These approaches align with current evidence highlighting the importance of gradual, personalized, and context-sensitive strategies in obesity management [6,22,29].

From a clinical perspective, the proposed framework provides a practical structure that can be implemented across different healthcare settings, in line with current pediatric obesity guidelines [5]. From a public health perspective, it underscores the need for coordinated actions that support healthy behaviors at multiple levels, as recommended in global strategies for obesity prevention [2].

## 5. Limitations

This study has several limitations. As a narrative review, it does not follow a systematic methodology, and the selection of literature may be subject to bias. Additionally, the proposed framework is based partly on clinical experience and requires further empirical validation.

In addition, the resource requirements and cost-effectiveness of implementing the proposed framework across different healthcare systems remain unknown and should be evaluated in future implementation studies [32].

Future research should focus on evaluating the effectiveness of system-oriented dietary counselling models in different populations and settings [10].

The proposed framework should be regarded as a conceptual and implementation-oriented model rather than an empirically tested intervention protocol. Its feasibility, acceptability, and effectiveness should be evaluated in future studies, including pilot interventions in diverse family structures and socioeconomic contexts. Comparative studies against conventional dietary counselling would be particularly valuable to determine whether the system-oriented approach improves adherence, behavioral outcomes, and weight-related indicators [32].

## 6. Conclusions

Dietary counselling in pediatric obesity should extend beyond individual nutrition advice and address the family, home, school, peer, community, and policy environments in which eating behaviors are formed and maintained. The proposed system-oriented framework offers a practical structure for integrating dietary assessment, patient education, collaborative goal setting, behavioral strategies, and environmental modification within routine counselling. By making contextual barriers explicit, the framework may help clinicians tailor recommendations to diverse family structures and resource settings. Future empirical studies should evaluate its feasibility, acceptability, and effectiveness compared with conventional counselling approaches.

## Figures and Tables

**Table 1:** Proposed structured visit-based dietary counselling model for pediatric obesity management.

Stage	Main Aim	Suggested Tools	Target Person	Measures/Outputs
Visit 1	Rapport, baseline assessment	24 h recall, brief dietary screener, family meal pattern interview	Child + caregiver(s)	BMI z-score, waist circumference, meal frequency, sugary drinks, screen time, sleep, PA level
Visit 2	Home food environment	Home food availability checklist, shopping routine discussion	Caregiver(s)	Availability of ultra-processed foods, fruit/vegetable access, meal routines
Visit 3	Dietary education	Plate model, food-based guidelines, label-reading exercise	Child + caregiver(s)	One or two SMART goals, e.g., beverages, breakfast, snacks
Visit 4	School and peer context	School eating questionnaire, pocket money/food purchase questions	Child + caregiver(s)	School meals, vending/sklepik use, peer-related eating
Visit 5	Physical activity and lifestyle	PA preference interview, sedentary behavior assessment, weekly movement plan	Child + caregiver(s)	Daily movement, sedentary time, enjoyable family-based activities, sleep routine, active transport opportunities
Follow-up every 4–6 weeks	Reinforcement and adaptation	Goal review, self-monitoring, problem-solving	Child + caregiver(s)	Goal adherence, barriers, new/modified SMART goals

## Data Availability

This article is a narrative review and does not report original research data. All data discussed are derived from previously published studies, which have been appropriately cited. Therefore, data sharing is not applicable.

## Abbreviations

The following abbreviations are used in this manuscript:

BMI	Body mass index
FBT	Family-Based Behavioral Treatment
PA	Physical activity
SMART	Specific, Measurable, Achievable, Relevant, and Time-bound
CBT	Cognitive–behavioral therapy
FFQ	Food Frequency Questionnaire

## References

[1] CDC Childhood Obesity Facts. https://www.cdc.gov/obesity/childhood-obesity-facts/childhood-obesity-facts.html.

[2] WHO World Health Organization (2016). Report of the Commission on Ending Childhood Obesity.

[3] Kawecka P., Kostecka M. (2024). The Role of the Family Environment and Parental Nutritional Knowledge in the Prevention of Behavioral Feeding Disorders in Toddlers and Preschool Children—A Narrative Review. J. Health Inequal..

[4] Smith J.D., Fu E., Kobayashi M. (2020). Prevention and Management of Childhood Obesity and Its Psychological and Health Comorbidities. Annu. Rev. Clin. Psychol..

[5] Hampl S.E., Hassink S.G., Skinner A.C., Armstrong S.C., Barlow S.E., Bolling C.F., Avila Edwards K.C., Eneli I., Hamre R., Joseph M.M. (2023). Clinical Practice Guideline for the Evaluation and Treatment of Children and Adolescents with Obesity. Pediatrics.

[6] Epstein L.H., Wilfley D.E., Kilanowski C., Quattrin T., Cook S.R., Eneli I.U., Geller N., Lew D., Wallendorf M., Dore P. (2023). Family-Based Behavioral Treatment for Childhood Obesity Implemented in Pediatric Primary Care: A Randomized Clinical Trial. JAMA.

[7] Swinburn B.A., Sacks G., Hall K.D., McPherson K., Finegood D.T., Moodie M.L., Gortmaker S.L. (2011). The Global Obesity Pandemic: Shaped by Global Drivers and Local Environments. Lancet.

[8] The Ecology of Human Development. https://www.hup.harvard.edu/books/9780674224575.

[9] Bandura A., Walters R.H. (1977). Social Learning Theory.

[10] Sukhera J. (2022). Narrative Reviews: Flexible, Rigorous, and Practical. J. Grad. Med. Educ..

[11] Birch L.L., Fisher J.O. (1998). Development of Eating Behaviors among Children and Adolescents. Pediatrics.

[12] Savage J.S., Fisher J.O., Birch L.L. (2007). Parental Influence on Eating Behavior: Conception to Adolescence. J. Law Med. Ethics J. Am. Soc. Law Med. Ethics.

[13] Maia C., Braz D., Fernandes H.M., Sarmento H., Machado-Rodrigues A.M. (2025). The Impact of Parental Behaviors on Children’s Lifestyle, Dietary Habits, Screen Time, Sleep Patterns, Mental Health, and BMI: A Scoping Review. Children.

[14] Shloim N., Edelson L.R., Martin N., Hetherington M.M. (2015). Parenting Styles, Feeding Styles, Feeding Practices, and Weight Status in 4–12 Year-Old Children: A Systematic Review of the Literature. Front. Psychol..

[15] Munawar K., Mukhtar F., Roy M., Majeed N., Jalaludin M.Y. (2024). A Systematic Review of Parenting and Feeding Practices, Children’s Feeding Behavior and Growth Stunting in Asian Countries. Psychol. Health Med..

[16] Mahmood L., Flores-Barrantes P., Moreno L.A., Manios Y., Gonzalez-Gil E.M. (2021). The Influence of Parental Dietary Behaviors and Practices on Children’s Eating Habits. Nutrients.

[17] Gupta S., Lal P., Sharma R., Gupta A., Chaudhary B.R. (2025). Systematic Review of Parental Influence on Pediatric Obesity: Exploring Dietary Habits, Physical Activity, and Intervention Strategies. Obes. Pillars.

[18] Llewellyn C., Wardle J. (2015). Behavioral Susceptibility to Obesity: Gene-Environment Interplay in the Development of Weight. Physiol. Behav..

[19] Glanz K., Sallis J.F., Saelens B.E., Frank L.D. (2005). Healthy Nutrition Environments: Concepts and Measures. Am. J. Health Promot. AJHP.

[20] Liu Y., Yang Z., Deng T., Bi C., Li H., Qu P., Chen Y., Liang D., Xu J., Li N. (2026). Food Environment and Childhood Overweight/Obesity: A Systematic Review. Adv. Nutr..

[21] Boutelle K.N., Kang Sim D.E., Rhee K.E., Manzano M., Strong D.R. (2021). Family-Based Treatment Program Contributors to Child Weight Loss. Int. J. Obes..

[22] Prochaska J.O., DiClemente C.C. (1983). Stages and Processes of Self-Change of Smoking: Toward an Integrative Model of Change. J. Consult. Clin. Psychol..

[23] Integrated Brief Interventions for Noncommunicable Disease Risk Factors in Primary Care: The Manual: BRIEF Project. https://www.who.int/europe/publications/i/item/9789289058551.

[24] Callahan E.A., National Academies of Sciences, Engineering, and Medicine, Health and Medicine Division, Food and Nutrition Board (2022). Methods for Dietary Assessment in Children 6 to 11 Years of Age. Approaches to Assessing Intake of Food and Dietary Supplements in Pregnant Women and Children 2 to 11 Years of Age: Proceedings of a Workshop Series.

[25] Thompson F.E., Subar A.F. (2017). Dietary Assessment Methodology. Nutrition in the Prevention and Treatment of Disease.

[26] Shahdan S., Sidek S. (2025). The Influence of Family Characteristics on Food Parenting Practices among Parents with School-Age Children and Adolescents: A Systematic Review. Appetite.

[27] MyPlate|Nutrition and Dietetics|Research Starters|EBSCO Research. https://www.ebsco.com.

[28] Gibbs H., Chapman-Novakofski K. (2013). Establishing Content Validity for the Nutrition Literacy Assessment Instrument. Prev. Chronic. Dis..

[29] Michie S., Richardson M., Johnston M., Abraham C., Francis J., Hardeman W., Eccles M.P., Cane J., Wood C.E. (2013). The Behavior Change Technique Taxonomy (v1) of 93 Hierarchically Clustered Techniques: Building an International Consensus for the Reporting of Behavior Change Interventions. Ann. Behav. Med. Publ. Soc. Behav. Med..

[30] Salvy S.-J., de la Haye K., Bowker J.C., Hermans R.C.J. (2012). Influence of Peers and Friends on Children’s and Adolescents’ Eating and Activity Behaviors. Physiol. Behav..

[31] Bull F.C., Al-Ansari S.S., Biddle S., Borodulin K., Buman M.P., Cardon G., Carty C., Chaput J.-P., Chastin S., Chou R. (2020). World Health Organization 2020 Guidelines on Physical Activity and Sedentary Behaviour. Br. J. Sports Med..

[32] Skivington K., Matthews L., Simpson S.A., Craig P., Baird J., Blazeby J.M., Boyd K.A., Craig N., French D.P., McIntosh E. (2021). A New Framework for Developing and Evaluating Complex Interventions: Update of Medical Research Council Guidance. BMJ.

[33] FAO (2019). FAO School Food and Nutrition Framework.

[34] Thomas T.W., Cankurt M. (2024). Influence of Food Environments on Dietary Habits: Insights from a Quasi-Experimental Research. Foods.

[35] Pont S.J., Puhl R., Cook S.R., Slusser W., Section On Obesity, Obesity Society (2017). Stigma Experienced by Children and Adolescents with Obesity. Pediatrics.

[36] WHO European Regional Obesity Report 2022. https://www.who.int/europe/publications/i/item/9789289057738.

[37] Roberto C.A., Swinburn B., Hawkes C., Huang T.T.-K., Costa S.A., Ashe M., Zwicker L., Cawley J.H., Brownell K.D. (2015). Patchy Progress on Obesity Prevention: Emerging Examples, Entrenched Barriers, and New Thinking. Lancet.

[38] Set of Recommendations on the Marketing of Foods and Non-Alcoholic Beverages to Children. https://www.who.int/publications/i/item/9789241500210.

[39] Fiscal Policies for Diet and the Prevention of Noncommunicable Diseases. https://www.who.int/publications/i/item/9789241511247.

[40] Darmon N., Drewnowski A. (2008). Does Social Class Predict Diet Quality?. Am. J. Clin. Nutr..

[41] World Health Organization (2012). Population-Based Approaches to Childhood Obesity Prevention.

[42] Waters E., de Silva-Sanigorski A., Hall B.J., Brown T., Campbell K.J., Gao Y., Armstrong R., Prosser L., Summerbell C.D. (2011). Interventions for Preventing Obesity in Children. Cochrane Database Syst. Rev..

